# Recent trends, risk factors, and disparities in low birth weight in California, 2005–2014: a retrospective study

**DOI:** 10.1186/s40748-018-0084-2

**Published:** 2018-08-08

**Authors:** Anura W. G. Ratnasiri, Steven S. Parry, Vivi N. Arief, Ian H. DeLacy, Laura A. Halliday, Ralph J. DiLibero, Kaye E. Basford

**Affiliations:** 1 Department of Health Care Services, Benefits Division, 1501 Capitol Ave, Suite 71.4104, MS 4600, P.O. Box 997417, Sacramento, CA 95899-7417 USA; 2 Department of Health Care Services, Clinical Assurance and Administrative Support Division, 1501 Capitol Ave, Sacramento, CA 95899-7417 USA; 3 School of Agriculture and Food Sciences, Faculty of Science, The University of Queensland, Brisbane, Qld 4072 Australia; 4 School of Biomedical Sciences, Faculty of Medicine, The University of Queensland, Brisbane, Qld 4072 Australia

**Keywords:** Low birth weight, Preterm birth, Prenatal care, Advance maternal age, Maternal health, Health behavior, Small for gestational age

## Abstract

**Background:**

Low birth weight (LBW) is a leading risk factor for infant morbidity and mortality in the United States. There are large disparities in the prevalence of LBW by race and ethnicity, especially between African American and White women. Despite extensive research, the practice of clinical and public health, and policies devoted to reducing the number of LBW infants, the prevalence of LBW has remained unacceptably and consistently high. There have been few detailed studies identifying the factors associated with LBW in California, which is home to a highly diverse population. The aim of this study is to investigate recent trends in the prevalence of LBW infants (measured as a percentage) and to identify risk factors and disparities associated with LBW in California.

**Methods:**

A retrospective cohort study included data on 5,267,519 births recorded in the California Birth Statistical Master Files for the period 2005–2014. These data included maternal characteristics, health behaviors, information on health insurance, prenatal care use, and parity. Logistic regression models identified significant risk factors associated with LBW. Using gestational age based on obstetric estimates (OA), small for gestational age (SGA), appropriate for gestational age (AGA) and large for gestational age (LGA) infants were identified for the periods 2007–2014.

**Results:**

The number of LBW infants declined, from 37,603 in 2005 to 33,447 in 2014. However, the prevalence of LBW did not change significantly (6.9% in 2005 to 6.7% in 2014). The mean maternal age at first delivery increased from 25.7 years in 2005 to 27.2 years in 2014. The adjusted odds ratio showed that women aged 40 to 54 years were twice as likely to have an LBW infant as women in the 20 to 24 age group. African American women had a persistent 2.4-fold greater prevalence of having an LBW infant compared with white women. Maternal age was a significant risk factor for LBW regardless of maternal race and ethnicity or education level. During the period 2017–2014, 5.4% of the singleton births at 23–41 weeks based on OE of gestational age were SGA infants (preterm SGA + term SGA). While all the preterm SGA infants were LBW, both preterm AGA and term SGA infants had a higher prevalence of LBW.

**Conclusions:**

In California, during the 10 years from 2005 to 2014, there was no significant decline in the prevalence of LBW. However, maternal age was a significant risk factor for LBW regardless of maternal race and ethnicity or education level. Therefore, there may be opportunities to reduce the prevalence of LBW by reducing disparities and improving birth outcomes for women of advanced maternal age.

**Electronic supplementary material:**

The online version of this article (10.1186/s40748-018-0084-2) contains supplementary material, which is available to authorized users.

## Background

The terminology currently used to describe infants with a low birth weight (LBW) for a given gestational age varies, including the terms small for gestational age, intrauterine growth restriction, and fetal growth restriction (FGR) [1, 2]. Small for gestational age is defined as a birth weight below the tenth percentile for gestational age [1]. However, some infants with a birth weight below the tenth percentile are normal, and their low weight is due to maternal constitutional factors including weight, height, parity, and ethnicity. These infants do not necessarily have an increased risk for perinatal morbidity and mortality [1, 2]. The term FGR is defined as an antepartum estimated fetal weight less than the tenth percentile for gestational age and its presence may be due to genetic or environmental factors. Most infants with FGR are born small for gestational age. Moderate FGR is defined as a birth weight in the third to tenth percentile, and severe FGR is defined as a birth weight less than the third percentile [1, 2].

The prevalence of LBW is greater in resource-limited countries. While current data show that up to 10% of term infants in developed countries have LBW, that figure is 20% in developing countries [3]. A recent report indicates that 19% of infants in resource-limited areas are born with LBW, and 22% of reported neonatal deaths occur in infants with LBW [3]. In 2012, the Child Health Epidemiology Reference Group evaluated 14 birth cohorts and applied the birth weight standards specified by the International Fetal and Newborn Growth Consortium for the twenty-first Century (INTERGROWTH-21st). Using this definition, LBW was found in 19.3% of live births in low-income and middle-income countries, and 22% of neonatal deaths occurred in infants born small for gestational age [3].

Perinatal mortality increases in infants with LBW, whether they are born at term or preterm [2, 4, 5]. Perinatal mortality increases as birth weight decreases, as shown in a recently published population-based study from Canada, where the highest infant mortality was found in infants with a birth weight less than the fifth percentile, for both term and preterm infants [5]. There are several factors that contribute to increased morbidity and mortality in LBW infants, including congenital malformations, cardiac and respiratory disorders, and perinatal asphyxia [2]. According to the INTERGROWTH-21st standards, infants with LBW are those born weighing less than 2500 g. Intrauterine growth restriction and preterm birth are often associated with LBW. Outcomes associated with LBW include short-term fetal or neonatal morbidity, including respiratory distress syndrome and necrotizing enterocolitis; long-term morbidity, including blindness and cerebral palsy; and early neonatal and infant mortality [6].

In 1995, David Barker first proposed that the fetal environment and early infant health status permanently program the development of the individual into old age, a theory known as the Barker hypothesis, or “fetal origins of adult disease” [7, 8]. Several epidemiological studies have confirmed that LBW is associated not only with developmental issues in surviving infants [9], but with the development of chronic conditions or diseases in adulthood, including coronary artery disease [9–13], stroke, reduced bone mass, dyslipidemia, hypertension, type II diabetes mellitus [14], cancer, osteoporosis, and psychiatric illnesses [8, 15–18]. Despite extensive research devoted to reducing the number of LBW infants, as well as policy statements and clinical and public health practices with the same aim, the prevalence of LBW in the United States has remained unacceptably and consistently high. In addition, racial and ethnic disparities in birth outcomes are well documented in the United States.

Life course health development models may be used to improve public health outcomes, including in the maternal and child health community [19]. As Pies and Kotelchuck (2014) described, there is a need for a framework to address the social determinants and causes of health inequalities and the current facilitators of disparities in maternal and infant health [20, 21].

The aim of this study is to examine the current trends in the prevalence of LBW in California, using the Birth Statistical Master Files (BSMF) compiled by the California Department of Public Health (CDPH), and to identify significant predictors of LBW and racial and ethnic disparities in LBW. By identifying these risk factors and disparities, intervention strategies can be developed to reduce the prevalence of LBW and improve the health of the general population, now and into the future.

## Methods

### Data sources and study design

We consulted the BSMF, compiled by the CDPH, for the period 2005 through 2014. The study was approved by the California Committee for the Protection of Human Subjects (Protocol ID: 16–10-2759) and the CDPH Vital Statistics Advisory Committee.

In this retrospective cohort study, we collected data on Californian resident births in the years 2005 to 2014. Descriptive statistics were used to characterize all resident births and the prevalence of LBW each year, according to maternal characteristics and perinatal health behavior variables obtained from the BSMF data set.

### LBW as a response variable

The response variable in this study was LBW infants. Birth weight was obtained from the birth files and coded as a dichotomous variable to indicate whether the infant was LBW (< 2500 g). Data cleaning was performed before analysis to exclude births with missing information on birth weight and those with any out-of-range values. There were 877 excluded birth records out of 5,267,519 resident births from 2005 to 2014.

### Explanatory maternal variables

The explanatory variables considered were maternal sociodemographic status, prenatal health behavior, health insurance status, prenatal care use during the first trimester, and parity [22]. Maternal sociodemographic characteristics included maternal age, education, race and ethnicity, and place of birth and residence. Prenatal health behaviors included smoking during both first and second trimesters and maternal prepregnancy body mass index (BMI). Obesity is commonly classified according to BMI, which is calculated as the individual’s weight in kilograms divided by their height in meters squared (kg/m^2^). Using the criteria of the World Health Organization, underweight is classified as a BMI < 18.5 kg/m^2^, normal weight as 18.5 to 24.9 kg/m^2^, overweight as 25.0 to 29.9 kg/m^2^; obesity class I as 30.0 to 34.9 kg/m^2^, obese obesity class II as 35.0 to 39.9, kg/m^2^ and obesity class III as ≥ 40 kg/m^2^ [23]. The type of health insurance was divided into public (Medi-Cal) and private, considered crude predictors of low and high income, respectively.

### Relationship between birth weight and gestational age on fetal growth

Gestational age affects fetal growth and birth weight can be categorized as small for gestational age (SGA) (< 10th percentile), appropriate for gestational age (AGA) (10th to 90th percentile), and large for gestational age (LGA) (> 90th percentile), using new gender specific intrauterine growth curves based on United States data by Olsen et al. (2010) [24].

We extended these three categories to six categories by characterizing gestational age of 23–41 weeks for singleton births as preterm (< 37 weeks of gestation) and term (≥ 37 weeks of gestation) births: preterm SGA, preterm AGA, preterm LGA, term SGA, term AGA, and term LGA [3]. The preterm AGA and term SGA groups were further extended into with and without LBW (< 2500 g) infants.

### Statistical analysis

To identify significant risk factors among the maternal characteristics and perinatal maternal health behaviors associated with LBW, we performed both unadjusted and adjusted logistic regression analysis. The adjusted analysis used multivariable logistic regression, controlling for potential confounding variables in maternal and perinatal health behaviors.

The analysis was extended to study the prevalence of LBW based on all births according to two different interaction scenarios: first, between maternal age and maternal race and ethnicity; and second, between maternal education level and maternal race and ethnicity. Finally, multivariate logistic regression modeling approaches were used to study these two interaction scenarios after controlling for appropriate confounding variables. Multivariate modeling was stratified by maternal age and maternal education level to elaborate disparities in race and ethnicity and to identify the high-risk subgroups. The reference groups for maternal age, race and ethnicity, and education level were the age group 20–24 years, white race, and an education level of a bachelor’s degree or higher, respectively. Calculated adjusted odds ratios (AORs) with 95% confidence intervals (95% CI) and *p* values are presented in the tables.

The logistic regression models were restricted to singleton births. The significance level was set at *p* = 0.05. All analyses were conducted using SAS, version 9.3 (SAS Institute Inc., Cary, NC, USA).

## Results

The descriptive statistics for the 5,267,519 resident births that occurred in California during the 10-year period from 2005 to 2014 are listed in Additional file 1: Table S1 in the Supplementary Materials. During this period, the mean maternal age for primiparous women increased by 1.5 years, from 25.7 years in 2005 to 27.2 years in 2014.

Table 1 shows the prevalence of LBW, according to each variable evaluated. Although the number of LBW infants decreased, from 37,603 in 2005 to 33,447 in 2014, the prevalence of LBW did not change significantly during the study period (6.9% in 2005 to 6.7% in 2014).

**Table 1:** Total number of low birth weight infants and prevalence according to maternal characteristics and perinatal health behaviors in California for the period 2005–2014

Year	2005	2006	2007	2008	2009	2010	2011	2012	2013	2014
Number of low birth weight infants	37,603	38,460	38,867	37,580	35,774	34,624	33,956	33,657	33,718	33,447
Percentage of low birth weight infants	6.9	6.8	6.9	6.8	6.8	6.8	6.8	6.7	6.8	6.7
Variable										
Maternal age (years)										
< 20	7.7	7.4	7.6	7.5	7.4	7.5	7.3	7.1	7.4	7.2
20–24	6.3	6.2	6.3	6.2	6.1	6.2	6.2	6.2	6.4	6.4
25–29	6.2	6.1	6.1	6.0	6.1	6.0	6.0	6.0	6.1	5.9
30–34	6.6	6.7	6.7	6.6	6.6	6.6	6.5	6.5	6.6	6.4
35–39	7.9	7.9	8.0	8.0	7.8	7.8	7.9	7.7	7.7	7.6
40–54	11.3	11.2	11.0	11.4	11.2	11.1	11.0	10.4	10.9	10.5
Maternal race and ethnicity										
Hispanic	6.2	6.3	6.3	6.1	6.2	6.2	6.2	6.1	6.4	6.3
White	6.5	6.3	6.4	6.4	6.2	6.1	6.1	5.7	6.0	5.7
Asian	7.6	7.7	7.6	7.8	8.1	7.9	7.9	7.9	7.7	7.2
Pacific Islander	7.2	7.5	6.9	6.9	6.3	7.1	7.3	6.5	6.4	6.6
African American	12.8	12.2	12.1	12.4	12.0	12.2	11.8	12.0	11.7	11.8
Multiple race	7.8	7.6	7.8	7.0	7.6	7.7	7.1	7.5	7.3	7.6
American Indian	6.6	6.7	7.5	7.6	6.4	6.8	6.2	6.6	7.0	6.3
Other/unknown	9.1	9.6	10.2	10.0	9.3	10.0	10.1	9.8	10.2	10.0
Maternal education level										
Less than high school diploma	6.6	6.7	6.6	6.5	6.6	6.7	6.6	6.5	6.8	6.9
High school diploma	7.0	6.8	6.7	6.8	6.6	6.6	6.7	6.5	6.7	6.6
Some college or associate degree	6.9	6.9	7.0	6.9	6.7	6.9	6.8	6.7	6.9	6.7
Bachelor’s degree or higher	6.8	6.9	7.0	6.9	6.9	6.7	6.6	6.6	6.5	6.2
Unknown	8.2	8.7	8.6	9.0	8.6	8.6	8.9	8.7	9.1	8.8
Maternal nativity										
Foreign-born	6.2	6.3	6.4	6.3	6.4	6.4	6.5	6.5	6.6	6.5
United States-born	7.4	7.3	7.3	7.3	7.1	7.1	7.0	6.8	6.9	6.8
Maternal demographic region										
Central Coast	6.4	6.3	6.1	6.2	6.0	5.8	6.1	5.9	6.2	6.0
Greater Bay Area	6.8	6.9	6.7	6.7	6.8	6.8	6.9	6.9	7.0	6.7
Inland Empire	6.9	6.7	6.9	6.9	6.8	6.8	6.7	6.9	7.1	6.8
Los Angeles County	7.3	7.4	7.4	7.3	7.2	7.3	7.1	6.9	7.0	6.9
Northern and Sierra	6.4	5.9	6.1	6.0	5.4	5.7	6.0	5.8	6.4	6.3
Orange County	6.4	6.4	6.5	6.4	6.6	6.4	6.7	6.3	6.2	6.3
Sacramento area	7.1	7.2	7.1	6.7	6.8	7.2	7.0	6.9	6.8	7.1
San Diego area	6.7	6.5	6.9	6.6	6.6	6.4	6.4	6.3	6.4	6.4
San Joaquin Valley	6.7	6.8	6.8	7.0	6.9	7.0	6.9	6.8	7.1	6.9
Source of prenatal care payment										
Private	6.8	6.8	6.9	6.9	6.8	6.8	6.9	6.7	6.8	6.6
Medi-Cal	6.7	6.7	6.7	6.6	6.6	6.6	6.6	6.6	6.8	6.8
First trimester prenatal care initiation										
Yes	6.7	6.7	6.8	6.8	6.7	6.7	6.7	6.6	6.8	6.6
No	7.6	7.2	6.8	6.6	6.7	6.8	6.8	6.7	6.7	6.8
Parity										
Primiparous	7.2	7.1	7.2	7.1	7.1	7.1	7.1	7.0	7.1	6.9
Multiparous (2–5)	6.5	6.5	6.5	6.5	6.4	6.4	6.4	6.4	6.5	6.4
Multiparous (6–12)	11.1	10.3	10.4	10.4	10.3	10.4	9.9	9.7	10.3	10.5
Plurality										
Singleton births	5.2	5.2	5.3	5.2	5.2	5.3	5.2	5.2	5.3	5.1
Multiple births	56.9	57.1	56.8	56.0	55.4	54.6	54.7	53.8	53.6	52.7
Maternal smoking during both first and second trimesters										
No	N/A	N/A	6.8	6.7	6.7	6.7	6.7	6.6	6.7	6.6
Yes	N/A	N/A	11.3	11.2	11.6	11.4	11.9	12.3	12.5	12.6
Prepregnancy body mass index (kg/m^2^)										
Underweight (≤18.5)	N/A	N/A	9.5	9.7	9.7	9.5	9.3	8.8	9.2	9.2
Normal (18.5–24.9)	N/A	N/A	6.7	6.8	6.7	6.7	6.7	6.6	6.8	6.5
Overweight (25.0–29.9)	N/A	N/A	6.3	6.1	6.0	6.2	6.3	6.3	6.3	6.3
Obese I (30.0–34.9)	N/A	N/A	6.6	6.3	6.5	6.7	6.5	6.2	6.5	6.7
Obese II (35.0–39.9)	N/A	N/A	6.8	6.9	7.0	6.6	6.2	6.9	6.8	6.6
Obese III (≥ 40)	N/A	N/A	6.9	7.0	6.5	7.3	6.6	6.9	7.2	6.7

All values are given as percentages, except for the number of low birth weight infants

N/A = Maternal smoking status and the variables needed to compute prepregnancy body mass index were not recorded during 2005 or 2006

Both unadjusted and adjusted logistic regression analyses (Table 2) showed significant differences in the prevalence of LBW within each characteristic studied: maternal age, education level, race and ethnicity, place of birth and residence, demographic region, smoking status, prepregnancy BMI, source of perinatal care payment, first-trimester perinatal care, and parity (all *p* < 0.001).

**Table 2:** Crude and adjusted odds ratio of low birth weight singleton births according to maternal characteristics and perinatal health behaviors in California for the period 2005–2014

Variable	Crude odds ratio		Adjusted odds ratio	
	OR (95% CI)	*p* value^a^	AOR (95% CL)	*p* value^a^
Maternal age (years)				
< 20	1.28 (1.26–1.30)	< .001	1.04 (1.01–1.06)	0.001
25–29	0.90 (0.89–0.91)	< .001	1.09 (1.07–1.11)	< .001
30–34	0.92 (0.91–0.94)	< .001	1.25 (1.23–1.27)	< .001
35–39	1.08 (1.06–1.09)	< .001	1.54 (1.51–1.56)	< .001
40–54	1.41 (1.38–1.44)	< .001	2.01 (1.95–2.06)	< .001
20–24 (ref)	Ref		Ref	
Maternal race and ethnicity				
African American	2.57 (2.53–2.62)	< .001	2.41 (2.36–2.46)	< .001
American Indian	1.40 (1.30–1.50)	< .001	1.31 (1.21–1.43)	< .001
Asian	1.53 (1.51–1.55)	< .001	1.80 (1.76–1.83)	< .001
Hispanic	1.27 (1.26–1.29)	< .001	1.30 (1.28–1.32)	< .001
Multiple race	1.45 (1.40–1.49)	< .001	1.41 (1.36–1.45)	< .001
Other/unknown	1.63 (1.58–1.68)	< .001	1.52 (1.45–1.59)	< .001
Pacific Islander	1.40 (1.31–1.50)	< .001	1.50 (1.39–1.62)	< .001
White (ref)	Ref		Ref	
Maternal education level				
Less than high school diploma	1.29 (1.28–1.31)	< .001	1.36 (1.34–1.39)	< .001
High school diploma	1.25 (1.24–1.27)	< .001	1.29 (1.27–1.31)	< .001
Some college or associate degree	1.23 (1.22–1.25)	< .001	1.27 (1.25–1.29)	< .001
Unknown	1.46 (1.43–1.49)	< .001	1.34 (1.29–1.39)	< .001
Bachelor’s degree or higher (ref)	Ref		Ref	
Maternal nativity				
United States-born	1.06 (1.05–1.07)	< .001	1.15 (1.13–1.16)	< .001
Foreign-born (ref)	Ref		Ref	
Maternal demographic region				
Central Coast	0.98 (0.95–1.01)	0.246	1.06 (1.03–1.11)	0.001
Greater Bay Area	1.09 (1.06–1.13)	< .001	1.06 (1.02–1.09)	0.001
Inland Empire	1.16 (1.13–1.19)	< .001	1.16 (1.12–1.20)	< .001
Los Angeles County	1.21 (1.17–1.24)	< .001	1.15 (1.11–1.18)	< .001
Orange County	1.02 (0.99–1.05)	0.274	1.06 (1.02–1.10)	0.001
Sacramento area	1.07 (1.03–1.10)	< .001	1.07 (1.03–1.11)	< .001
San Diego area	1.04 (1.01–1.08)	0.006	1.10 (1.06–1.14)	< .001
San Joaquin Valley	1.21 (1.18–1.25)	< .001	1.20 (1.16–1.24)	< .001
Northern and Sierra	Ref		Ref	
Source of prenatal care payment				
Medi-Cal (Public)	1.17 (1.16–1.19)	< .001	1.13 (1.12–1.15)	< .001
Private insurance (ref)	Ref		Ref	
First trimester prenatal care initiation				
No	1.11 (1.10–1.13)	< .001	1.03 (1.02–1.04)	< .001
Yes (ref)	Ref		Ref	
Parity				
Primiparous	1.40 (1.39–1.42)	< .001	1.57 (1.55–1.58)	< .001
Multiparous (6–12)	1.67 (1.63–1.72)	< .001	1.20 (1.17–1.25)	< .001
Multiparous (2–5) (ref)	Ref		Ref	
Maternal smoking during both first and second trimesters				
Yes	2.11 (2.05–2.16)	< .001	1.98 (1.92–2.04)	< .001
No (ref)	Ref		Ref	
Maternal prepregnancy body mass index (kg/m^2^)				
Underweight (< 18.5)	1.58 (1.55–1.61)	< .001	1.49 (1.46–1.52)	< .001
Overweight (25.0–29.9)	0.95 (0.94–0.96)	< .001	0.95 (0.94–0.97)	< .001
Obese I (30.0–34.9)	1.00 (0.99–1.02)	0.919	0.99 (0.97–1.00)	0.118
Obese II (35.0–39.9)	1.04 (1.02–1.06)	< .001	1.01 (0.99–1.04)	0.315
Obese III (≥ 40)	1.08 (1.06–1.11)	< .001	1.02 (0.99–1.05)	0.237
Normal (18.5–24.9) (ref)	Ref		Ref	

*AOR* adjusted odds ratio, *BMI* body mass index, *CI* confidence interval, *OR* odds ratio

Ref = Reference group

^a^ *p* value determined using the χ^2^ test

### Maternal age

The prevalence of births in younger women declined over the study period (Additional file 1: Table S1). Births in women younger than 20 years decreased by 41%, from 9.3% in 2005 to 5.4% in 2014. In young adults (age 20–24 years), births decreased by 18%, from 22.9% in 2005 to 18.8% in 2014. In contrast, births in older women increased over the 10-year study period. Births in women aged 40–54 years increased by 20%, from 3.5% in 2005 to 4.3% in 2014; similar trends were observed for women in the age groups 30–34 years and 35–39 years. Table 1 shows the prevalence of LBW according to maternal characteristics and perinatal health behaviors for each year of the study period.

**Fig. 1:**
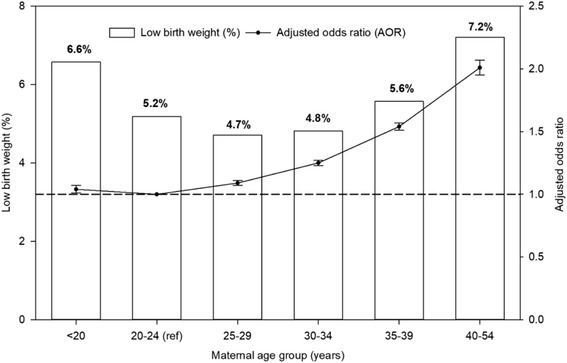
Unadjusted prevalence and adjusted odds ratios of low birth weight of singleton births by maternal age in California for the period 2005–2014

The unadjusted prevalence and AORs of LBW for singleton births by maternal age is presented in Fig. 1. Women in the oldest age group (40–54 years) were twice as likely to have an LBW infant than women in the 20–24 years reference age group (AOR, 2.01; 95% CI, 1.95–2.06). The 35–39 years age group was 54% more likely to have an LBW infant compared with the reference age group (AOR, 1.54; 95% CI, 1.51–1.56). Women in the 30–34 years age group had a 25% greater chance of having an LBW infant (AOR, 1.25; 95% CI, 1.23–1.27) (Fig. 1 and Table 2). Maternal age was a significant predictor of LBW in California.

### Maternal race and ethnicity

Almost 50% of births in California were to women of Hispanic ethnicity, followed by women who were White and Asian. Women of Asian ethnicity accounted for more than 32% of the increase in LBW births, from 11.3% in 2005 to 14.9% in 2014 (Additional file 1: Table S1).

In 2014, the overall prevalence of LBW was 6.7% (Table 1). However, 11.8% of infants born to African American women were LBW compared with 5.7% of those born to White women and 6.3% of those born to Hispanic women (Table 1). In 2014, the prevalence of LBW infants born to African American women was nearly twice that of White women and 88% greater than in Hispanic women. From 2005 to 2014, the prevalence of LBW births decreased at a slower rate in African American women (7.7%) compared with White women (12.3%).

As shown in Table 2, there were marked disparities in the prevalence of LBW infants born to women of different racial and ethnic groups. African American women had a persistent 2.4-fold prevalence of LBW infants throughout the study period compared with White women (AOR, 2.41; 95% CI, 2.36–2.46).

Compared with White women, Asian women were 80% more likely to give birth to an LBW infant (AOR, 1.80; 95% CI, 1.76–1.83). Pacific Islanders were 50% more likely to give birth to an LBW infant than White women (AOR, 1.50; 95% CI, 1.39–1.62), and Hispanic women were 30% more likely to give birth to an LBW infant than White women (AOR, 1.30; 95% CI, 1.28–1.32) (Table 2).

### Interaction between maternal age and maternal race and ethnicity

**Fig. 2:**
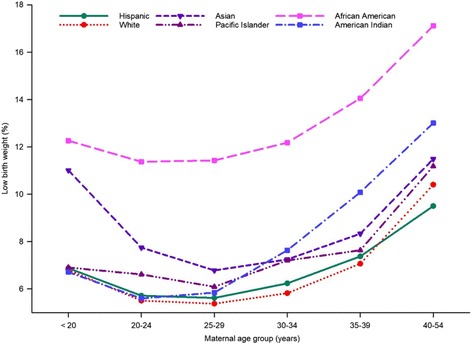
Unadjusted prevalence of low birth weight by maternal age and maternal race and ethnicity in California for the period 2005–2014

To identify the association between maternal age and race and ethnicity for LBW, we cross-tabulated the data for LBW according to these two variables. As shown in Fig. 2, the prevalence of LBW was not consistent across maternal age for racial and ethnic groups. African American women had a consistently higher prevalence of LBW compared with other races and ethnicities in each maternal age group. The wide gap in the prevalence of LBW between African American and White or Hispanic women was consistent for each age group (Fig. 2). Moreover, an almost equivalent higher observed prevalence of LBW was observed for Asian women in the youngest and oldest age groups, resulting in a U-shaped response (Fig. 2). All race and ethnic groups showed rising prevalence of LBW with increasing age, especially from 30 years of age (Fig. 2), but the rate of increase was greatest for American Indian women.

Additional file 1: Table S2 provides the adjusted odds ratios for maternal age for each racial and ethnic group. As indicated in the unadjusted prevalence (Fig. 2), the likelihood of having a LBW infant was greater with increasing maternal age, mostly from 30 years of age, with the highest prevalence for women in the age group 40–54 years. In contrast to other race and ethnic groups, Asian women were more likely to have LBW infants when they were younger, less than 20 years of age, compared with the reference group - women in the age group 20–24 years. American Indian women with a maternal age of 40–54 years were three times more likely to have LBW infants than the reference age group of 20–24 years (Additional file 1: Table S2).

### Maternal education level

In California, births in women with less than a complete high school education decreased by 40%, from 27.3% in 2005 to 16.3% in 2014. During the same period, births in women with a high school diploma as their highest level of education decreased by 12% (Additional file 1: Table S1).

Table 1 shows the prevalence of LBW according to maternal education level. The prevalence of LBW differed in women by education level, although these variations were smaller than those observed for differing age or racial-ethnic group (Table 1). Women with less than a high school diploma had a 36% greater chance (AOR, 1.36; 95% CI, 1.34–1.39) of having an LBW infant than the reference group of women with a bachelor’s degree or higher (Table 2).

### Interaction between maternal education level and race and ethnicity

**Fig. 3:**
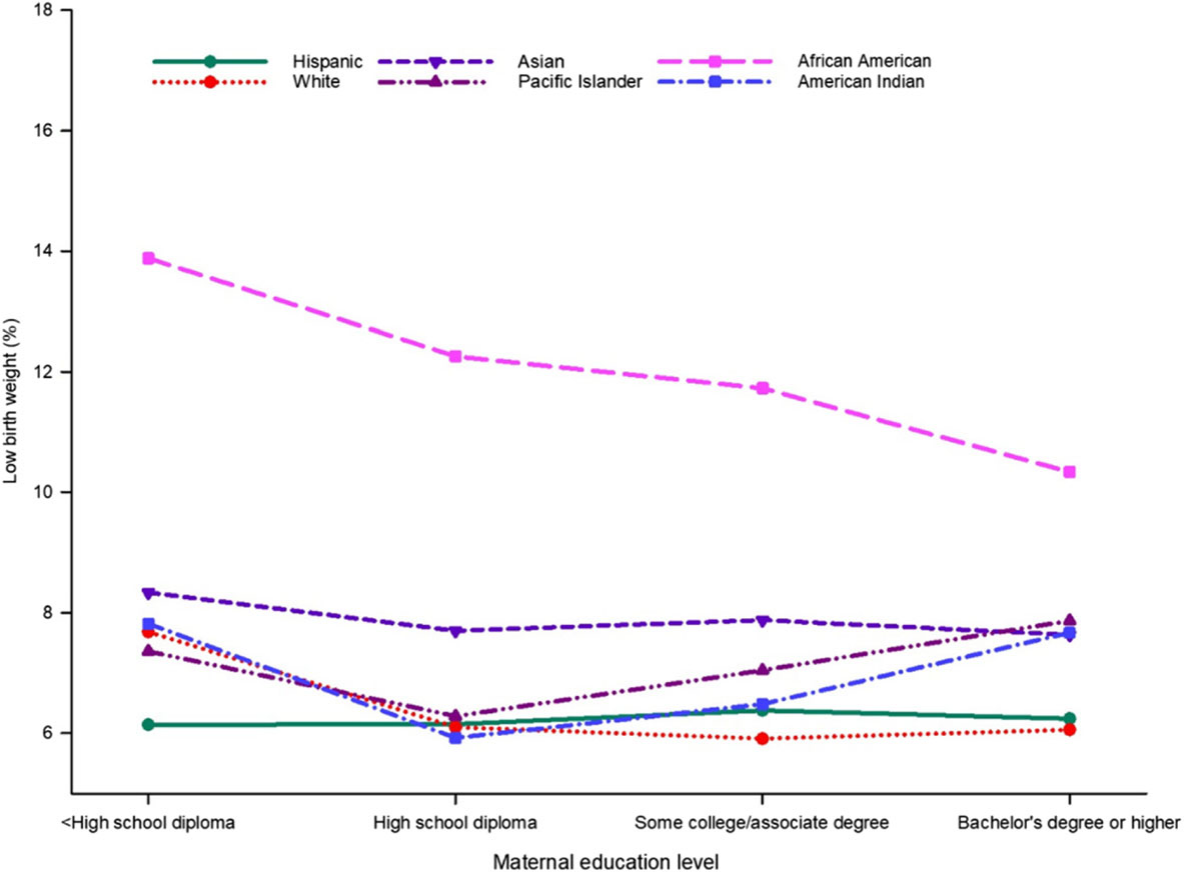
Unadjusted prevalence of low birth weight by maternal education level and maternal race and ethnicity in California for the period 2005–2014

To elaborate on the differences in LBW prevalence between maternal educational levels and race and ethnicity, we cross-tabulated the data for LBW accordingly. As shown in Fig. 3, the prevalence of LBW from lower to higher educational levels differed across racial and ethnic groups (Fig. 2). Unadjusted LBW prevalence was quite similar for women of Hispanic ethnicity, regardless of their educational level, but the magnitude of the disparity varied for other races (Fig. 3). African American women of all education levels had a higher unadjusted prevalence of LBW than women of every other race and education level. The higher prevalence of LBW was most prominent among African American women with a less than high school diploma. However, prevalence of LBW significantly declined with higher educational attainment, with the lowest prevalence among women with a bachelor’s degree or higher (Fig. 3).

Additional file 1: Table S3 presents adjusted odds ratios for maternal education level for each racial and ethnic group. Women in most race and ethnic groups with educational level less than a high school diploma were more likely to deliver LBW infants when compared with women having a bachelor’s degree or higher, but to a lesser extent for Asian and Pacific Islander women (Additional file 1: Table S3).

### Maternal place of birth

From 2005 to 2014, births to foreign-born women decreased by 18%, and births to United States-born women increased by almost 16% (Additional file 1: Table S1). Women born in the United States were 15% more likely (AOR, 1.15; 95% CI, 1.13–1.16) to have an LBW infant than were foreign-born women (Table 2).

### Maternal geographic region

Within each year, almost 26% of California births occurred in Los Angeles County, followed by the greater Bay Area region with slightly more than 17%, and San Joaquin Valley with about 13% of births in the state (Additional file 1: Table S1). Women in the San Joaquin Valley region were 20% more likely (AOR, 1.20; 95% CI, 1.16–1.24) to have an LBW infant compared with those in the Northern and Sierra regions (Table 2).

### Perinatal health behaviors

Maternal smoking during both first and second trimesters decreased significantly, by 31%, from 2007 to 2014 (Additional file 1: Table S1). Women who smoked during the first and second trimesters of pregnancy were almost twice as likely (AOR, 1.98; 95% CI, 1.92–2.04) to have an LBW infant than women who did not smoke (Table 2).

The prevalence of LBW births in women who were underweight or of normal weight, based on their prepregnancy BMI, decreased by 9.1 and 6.3%, respectively, from 2007 to 2014. However, the prevalence of LBW births to women who were in the obese I, obese II, and obese III categories increased by 12.0, 21.3, and 26.9%, respectively, from 2007 to 2014 (Table 1). While obesity did not increase the likelihood of LBW, underweight women (prepregnancy BMI < 18.5 kg/m^2^) were 49% more likely to have an LBW infant than were women of normal prepregnancy weight (AOR, 1.49; 95% CI, 1.46–1.52) (Table 2).

### Insurance type and first-trimester perinatal care

Consistent trends were observed for the percentages of births paid for by Medi-Cal and private insurance. The 2014 figures for California show that 52.7% of births were covered by private insurance and 47.3% were covered by Medi-Cal. Women dependent on Medi-Cal as their source of perinatal care payment were 13% more likely to have an LBW infant than women with private insurance (AOR, 1.13; 95% CI, 1.12–1.15) (Table 2). Overall, the use of first-trimester perinatal care decreased slightly, from 86.6% in 2005 to 83.2% in 2014.

### Birth characteristics

From 2005 to 2014, the prevalence of multiple births was consistent at 3.2% (Additional file 1: Table S1). Parity was consistent over the study period for each of the three groups considered: primiparous, multiparous with 2 to 5 deliveries, and multiparous with 6 to 12 deliveries (Additional file 1: Table S1). Women who were primiparous (AOR, 1.57; 95% CI, 1.55–1.58) or multiparous with 6 to 12 births (AOR, 1.20; 95% CI, 1.17–1.25) were more likely to have an LBW infant than multiparous women with 2 to 5 births (Table 2).

### Relationship between birth weight and gestational age on fetal growth

**Fig. 4:**
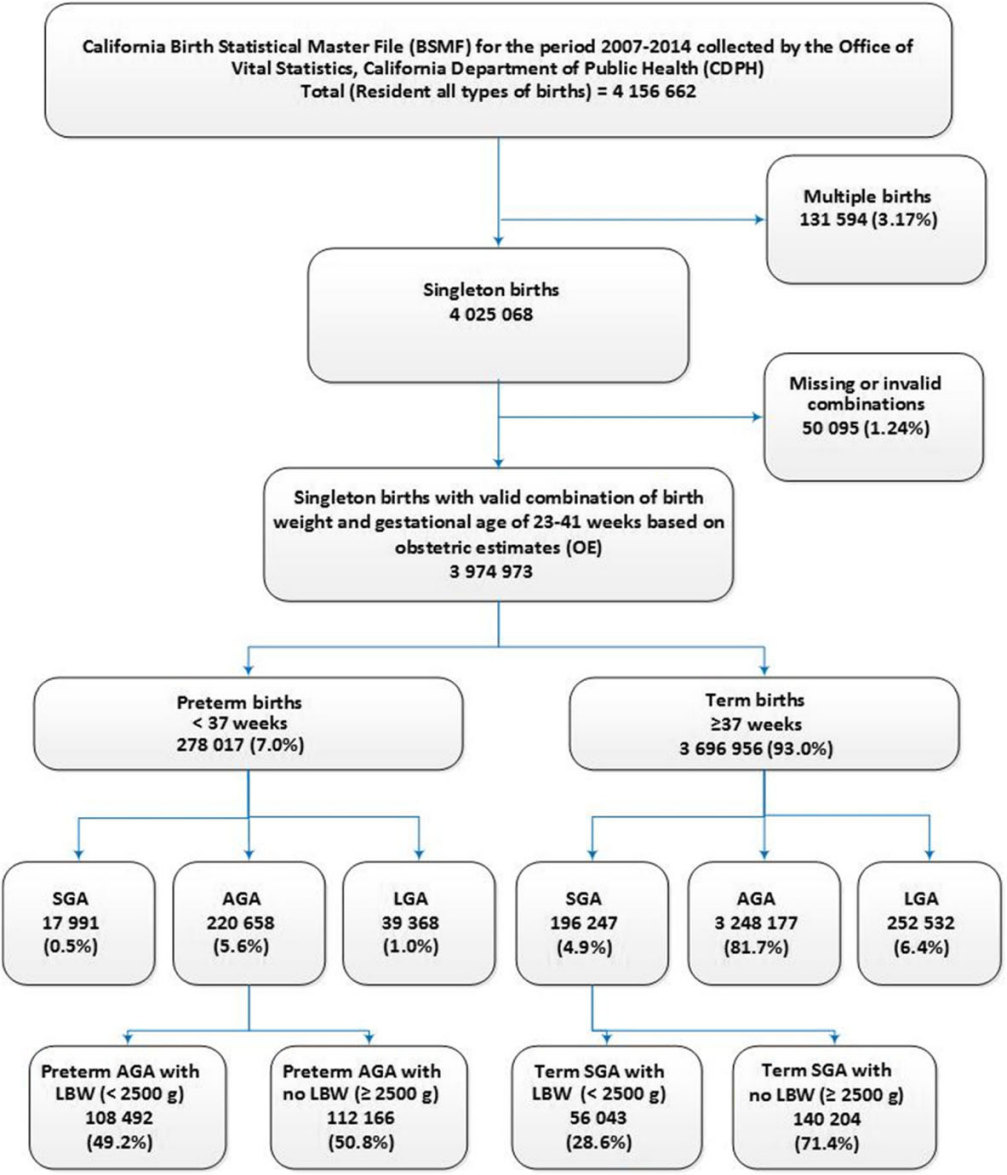
Distribution of singleton births at 23–41 weeks of gestation with respect to birth weight and gestational age based on obstetric estimates (OE) in California for the period 2007–2014. SGA: small for gestational age; *AGA* appropriate for gestational age, *LGA* large for gestational age, *LBW* low birth weight (< 2500 g)

Information on 3,974,973 singleton births in California for the period 2007–2014 was available to elaborate on the relationship between birth weight and gestational age based on OE (Fig. 4). For these births at 23–41 weeks, the 7% of preterm births (< 37 weeks of gestation) comprised of 0.5% preterm SGA, 5.6% preterm AGA, and 1.0% preterm LGA. Among preterm AGA, 49.2% of births were LBW infants while 100% of the preterm SGA births were LBW infants (Fig. 4).

Of the 93% of term births (≥ 37 weeks of gestation), 4.9% were term SGA births, 81.7% term AGA births, and 6.4% term LGA births. Among term SGA births, the prevalence of LBW was 28.6% (Fig. 4). Overall, 5.4% of these singleton births at 23 to 41 weeks based on OE of gestational age were SGA infants (preterm SGA + term SGA).

## Discussion

This retrospective cohort study, evaluating 5,267,519 resident births that occurred in California from 2005 to 2014, shows that the prevalence of LBW did not change significantly over that 10-year period.

Births to older women, aged from 30 to 54 years, increased over the study period, a trend that is consistent with the steadily increasing national mean maternal age since 2006 [22, 25–28]. The term “advanced maternal age” is used for women who are aged 35 years or greater at the time of delivery; advanced maternal age is considered a major risk factor for poorer pregnancy and perinatal outcomes [29, 30]. Sauer (2015) discussed the underlying reasons for the increased LBW prevalence in women of advanced maternal age [28]. The strong association between maternal age and birth weight reported by other studies was also found in our study [25, 31]. Women 35–39 years of age were more likely, and women aged 40–54 years were twice as likely, to have an LBW baby compared with women aged 20–24 years. Women aged less than 20 years and aged 40–54 years had a higher prevalence of LBW infants, regardless of their education level. However, Goisis et al. (2017) found that advanced maternal age is not independently associated with the risk of LBW or preterm delivery among women who have had at least 2 previous live births [30].

Disparities in the prevalence of LBW infants between racial and ethnic groups in the United States have been well documented [32, 33]. The persistence of a gap in LBW prevalence between African Americans and Whites is seen throughout the country and continues to be a serious public health problem (Table 1).

The findings of our study are consistent with those of previously published studies that have reported substantial disparities in the prevalence of LBW between women of different racial and ethnic groups. In our study, African American women had a more than 2-fold increase in the prevalence of LBW throughout the study period compared with White women (Table 2). The wide gap in the prevalence of LBW between African American and White or Hispanic women was consistent for each age group and across the 10-year period of the study.

During 2005, the prevalence of LBW in Hispanic women was 0.3% lower than in White women (6.2% vs. 6.5%). However, the prevalence of LBW was 0.6% greater in Hispanic births compared with births in White women (6.3% vs. 5.7%) in 2014. Therefore, given the increasing number of Hispanic births in California, the findings of this 10-year study provide an alert to the increasing gap in birth weights between Hispanic and White babies.

Overall, the prevalence of LBW when the mother is 40–54 years of age is double that when she is 20–24 years of age. This finding holds true for all groups except for Asian women and those a Multirace origin. Even at the highest education level, African American women had a greater prevalence of LBW compared with other ethnic groups, suggesting persistent disparities based on ethnicity.

The prevalence of United States adults who smoke cigarettes declined, from 20.9% in 2005 to 16.8% in 2014 [34]. Consistent with national studies, the number of women in our study who smoked tobacco during the first and second trimester decreased between 2007 and 2014 (Table 1). However, pregnant smokers have been reported to be almost twice as likely to have an LBW infant than nonsmokers [35]. The latest United States Surgeon General’s Report on Smoking and Health states that tobacco use during pregnancy remains a major preventable cause of disease and death of the mother, fetus, and infant [36]. Women who smoke during pregnancy are more likely to deliver LBW babies, even if the pregnancy is carried to full term.

Data on maternal smoking and prepregnancy height and weight have been collected in California only since 2007; this study is the first to report trends in prepregnancy BMI. The results of our study provide population-based information on BMI for women of childbearing age. The prevalence of births in women who were underweight or of normal weight, based on prepregnancy BMI, decreased during the study period, but the prevalence of births to women who were in the obese I, obese II, and obese III categories increased significantly (Table 2). Consistent with previous studies, ours found that underweight women are more likely to have an LBW infant than women with a normal prepregnancy weight [37, 38]. Our study found no significant association between prepregnancy obesity and the risk of having an LBW infant.

The rising prevalence of women in all three obesity classes in California is a public health concern for both women and children. According to a recent Institute of Medicine report, maternal obesity before, during, and after pregnancy poses serious health problems for both mothers and children [39]. Obesity contributes to gestational diabetes [40–42], preterm delivery [40, 42, 43], fetal injury during delivery, intrauterine mortality [44], and shorter duration of breastfeeding [45]. The long-term outcomes of maternal obesity include chronic disease such as diabetes, cardiovascular disease, and premature death. Obesity also carries an increased risk of adverse complications in the subsequent pregnancy for both mother and baby [40, 44, 46].

This study found significant differences in LBW according to the maternal place of birth and residence (Table 2). From 2005 to 2014, births in foreign-born women decreased from 46.6 to 38.1%, but they increased for United States-born women, from 53.4 to 61.9%. The former were less likely to deliver an LBW infant, a finding that has been reported in previous studies. In a study of mothers in New York City, foreign-born women had lower prevalence of LBW than did United States-born women [32]. Acevedo-Garcia et al. (2005) noted that the effect of being foreign-born on LBW differs according to maternal education and race and ethnicity [47].

We did not encounter any previously published studies that included maternal geographic region as a predictor of having an LBW infant. Our findings show that the prevalence of LBW differs in different regions of California (Table 2). Infants born in the San Joaquin Valley region are more likely to be of LBW compared with those born in the Northern and Sierra regions. Our study also shows that women who depend on Medi-Cal as their source of perinatal care payment, an indicator of lower socioeconomic status, are more likely to have an LBW infant than women who have private health insurance.

Alexander and Korenbrot (1995) reported on the role of perinatal care in preventing LBW. Our results confirm their finding, that attendance at perinatal care during the first trimester is associated with reduced LBW [48].

Maternal parity is a well-recognized predictor of infant birth weight; the lowest birth weights are found in infants born to primiparous women [49]. Our results confirm that parity is a significant predictor of LBW (Table 2). Primiparous and multiparous women with 6 to 12 prior deliveries were more likely to have an LBW infant than were multiparous women with 2 to 5 prior deliveries. This finding is consistent with a study by Hinkle et al. (2014), which found a nonlinear association in which birth weight increased up to parity of 4, then stabilized from parity of 4 to 7 [50].

Both preterm AGA and term SGA births demonstrated a high prevalence of LBW infants. Infants born SGA, whether term or preterm, carry a considerably higher risk of mortality and morbidity in the neonatal period and beyond when compared with AGA infants [3]. The risk is even greater among infants born both preterm and SGA [51].

There are several limitations to this study. Maternal characteristics were restricted to those contained within the BSMF compiled by the CDPH from 2005 to 2014. Maternal age, race and ethnicity, education level, smoking status during pregnancy (usually under-reported), and prepregnancy height and weight were self-reported. Despite these limitations and the inclusion of many possible confounding variables, our study demonstrates significant trends in LBW over a 10-year period in the highly diverse population of California and includes analysis of almost 5 million births.

## Conclusions

There was no significant decline in the prevalence of LBW during this 10-year period in California, but maternal age, race and ethnicity, education level, smoking status during pregnancy, and parity are significant risk factors for LBW. Therefore, there may be opportunities to reduce LBW by improving birth outcomes for women giving birth at an advanced maternal age, and by developing public health models to address the identified risk factors and improve the health of the population. The findings of this study illustrate the opportunities to improve fetal, infant, and adult health outcomes, not only in California but throughout the United States. Given the complexity of the etiology of LBW, further research is required on the genetic and epigenetic factors that interact with the social, ethnic, and age-related influences identified in this study.

## Additional file

Additional file 1: **Table S1.** Recorded births and percentage of births according to maternal characteristics and perinatal health behaviors in California for the period 2005–2014. **Table S2.** Likelihood of low birth weight listed as adjusted odds ratios (95% confidence intervals) for maternal age for each maternal race and ethnic group, after accounting for confounding effects in California for the period 2005–2014. **Table S3.** Likelihood of low birth weight listed as adjusted odds ratios (95% confidence intervals) for maternal education for each maternal race and ethnic group, after accounting for confounding effects in California for the period 2005–2014. (DOCX 52 kb)

## Abbreviations

95% CI: 95% confidence interval
AGA: Appropriate for gestational age
AOR: Adjusted odds ratio
BSMF: Birth Statistical Master Files
CDPH: California Department of Public Health
FGR: Fetal growth restriction
LBW: Low birth weight
LGA: Large for gestational age
OE: Obstetric estimates
SGA: Small for gestational age
