# Supplementary material for: Recent trends, risk factors, and disparities in low birth weight in California, 2005–2014: a retrospective study
Source: Matern Health Neonatol Perinatol. 2018 Aug 8;4:15. doi: 10.1186/s40748-018-0084-2 (PMC6081945; doi:10.1186/s40748-018-0084-2)
Supplement: Supplementary file 1 — Table S1. Recorded births and percentage of births according to maternal characteristics and perinatal health behaviors in California for the period 2005–2014. Table S2. Likelihood of low birth weight listed as adjusted odds ratios (95% confidence intervals) for maternal age for each maternal race and ethnic group, after accounting for confounding effects in California for the period 2005–2014. Table S3. Likelihood of low birth weight listed as adjusted odds ratios (95% confidence intervals) for maternal education for each maternal race and ethnic group, after accounting for confounding effects in California for the period 2005–2014. (DOCX 52 kb) [file 40748_2018_84_MOESM1_ESM.docx]

**Table S1. Recorded births and percentage of births according to maternal characteristics and perinatal health behaviors in California for the period 2005–2014**

| **Year** | **2005** | **2006** | **2007** | **2008** | **2009** | **2010** | **2011** | **2012** | **2013** | **2014** |
| --- | --- | --- | --- | --- | --- | --- | --- | --- | --- | --- |
| **Number of births** | **548 700** | **562 157** | **566 137** | **551 567** | **526 774** | **509 979** | **502 023** | **503 788** | **494 392** | **502 002** |
| **Mean maternal age at first delivery (years)** | **25.67** | **25.57** | **25.56** | **25.65** | **25.80** | **26.03** | **26.29** | **26.56** | **26.87** | **27.18** |
| ***Maternal age (years)*** |  |  |  |  |  |  |  |  |  |  |
| <20 | 9.3 | 9.5 | 9.6 | 9.5 | 9.2 | 8.5 | 7.7 | 7.0 | 6.2 | 5.4 |
| 20–24 | 22.9 | 23.0 | 22.6 | 22.2 | 21.6 | 21.1 | 20.5 | 20.1 | 19.6 | 18.8 |
| 25–29 | 26.2 | 26.4 | 26.6 | 26.7 | 26.8 | 26.8 | 26.8 | 26.6 | 26.3 | 26.4 |
| 30–34 | 24.4 | 23.7 | 23.9 | 24.1 | 24.5 | 25.3 | 26.4 | 27.2 | 28.1 | 29.0 |
| 35–39 | 13.8 | 13.8 | 13.9 | 14.0 | 14.1 | 14.3 | 14.5 | 15.0 | 15.5 | 16.2 |
| 40–54 | 3.5 | 3.6 | 3.5 | 3.7 | 3.8 | 3.9 | 4.1 | 4.2 | 4.2 | 4.3 |
| ***Maternal race and ethnicity*** |  |  |  |  |  |  |  |  |  |  |
| Hispanic | 51.5 | 52.2 | 52.5 | 52.1 | 51.3 | 50.5 | 49.7 | 48.6 | 48.2 | 47.2 |
| White^a^ | 28.4 | 27.4 | 26.7 | 26.5 | 26.8 | 27.6 | 27.7 | 27.2 | 27.9 | 27.5 |
| Asian^b^ | 11.3 | 11.2 | 11.7 | 11.8 | 11.9 | 11.9 | 12.3 | 13.6 | 13.8 | 14.9 |
| Pacific Islander^c^ | 0.5 | 0.5 | 0.5 | 0.5 | 0.5 | 0.4 | 0.4 | 0.4 | 0.4 | 0.4 |
| African American | 5.2 | 5.3 | 5.2 | 5.3 | 5.4 | 5.4 | 5.4 | 5.3 | 5.2 | 5.0 |
| Multiple races | 1.4 | 1.5 | 1.7 | 1.8 | 1.9 | 2.0 | 2.1 | 2.2 | 2.3 | 2.3 |
| American Indian^d^ | 0.4 | 0.4 | 0.4 | 0.4 | 0.4 | 0.4 | 0.4 | 0.4 | 0.4 | 0.3 |
| Other/unknown^e^ | 1.4 | 1.6 | 1.5 | 1.7 | 1.9 | 1.8 | 1.9 | 2.4 | 1.9 | 2.3 |
| ***Maternal education level*** |  |  |  |  |  |  |  |  |  |  |
| Less than high school diploma | 27.3 | 36.5 | 26.9 | 25.9 | 24.3 | 22.9 | 20.9 | 19.1 | 17.5 | 16.3 |
| High school diploma | 27.6 | 17.7 | 26.4 | 26.2 | 25.7 | 25.2 | 24.8 | 24.6 | 24.6 | 24.2 |
| Some college or associate degree | 18.6 | 20.9 | 21.1 | 21.9 | 22.7 | 23.7 | 24.8 | 25.0 | 25.7 | 26.0 |
| Bachelor's degree or higher | 23.9 | 22.0 | 22.3 | 22.8 | 23.7 | 24.8 | 25.6 | 26.7 | 27.9 | 29.3 |
| Unknown | 2.8 | 2.9 | 3.4 | 3.2 | 3.7 | 3.5 | 3.9 | 4.6 | 4.3 | 4.3 |
| ***Maternal nativity*** |  |  |  |  |  |  |  |  |  |  |
| Foreign-born | 46.6 | 46.5 | 45.9 | 44.6 | 42.9 | 41.2 | 39.8 | 39.4 | 38.0 | 38.1 |
| United States-born | 53.4 | 53.5 | 54.1 | 55.4 | 57.1 | 58.8 | 60.2 | 60.7 | 62.0 | 61.9 |
| ***Maternal demographic region*** |  |  |  |  |  |  |  |  |  |  |
| Central Coast | 6.0 | 5.9 | 5.9 | 6.0 | 5.9 | 6.0 | 6.0 | 5.8 | 5.9 | 5.8 |
| Greater Bay Area | 17.3 | 17.1 | 17.2 | 17.3 | 17.4 | 17.2 | 17.3 | 17.5 | 17.5 | 17.6 |
| Inland Empire | 11.8 | 12.2 | 12.3 | 12.1 | 12.1 | 12.2 | 12.2 | 12.1 | 12.2 | 12.2 |
| Los Angeles County | 27.4 | 27.0 | 26.8 | 26.8 | 26.5 | 26.1 | 26.0 | 26.1 | 26.0 | 25.9 |
| Northern and Sierra | 3.0 | 3.0 | 3.0 | 3.0 | 3.0 | 3.1 | 3.1 | 3.1 | 3.2 | 3.1 |
| Orange County | 8.0 | 7.9 | 7.8 | 7.7 | 7.7 | 7.5 | 7.6 | 7.6 | 7.5 | 7.7 |
| Sacramento area | 5.4 | 5.4 | 5.4 | 5.4 | 5.4 | 5.5 | 5.5 | 5.4 | 5.5 | 5.5 |
| San Diego area | 8.9 | 8.9 | 9.0 | 9.1 | 9.1 | 9.4 | 9.3 | 9.4 | 9.4 | 9.5 |
| San Joaquin Valley | 12.3 | 12.6 | 12.6 | 12.7 | 12.9 | 13.0 | 13.1 | 12.9 | 12.9 | 12.8 |
| ***Source of prenatal care payment*** |  |  |  |  |  |  |  |  |  |  |
| Private | 50.9 | 50.4 | 50.1 | 49.9 | 49.5 | 49.1 | 50.0 | 50.5 | 51.4 | 52.7 |
| Medi-Cal | 49.1 | 49.6 | 49.9 | 50.2 | 50.5 | 50.9 | 50.0 | 49.5 | 48.6 | 47.3 |
| ***First trimester prenatal care*** |  |  |  |  |  |  |  |  |  |  |
| Yes | 86.6 | 85.9 | 82.9 | 82.4 | 82.9 | 83.5 | 83.5 | 83.8 | 83.6 | 83.2 |
| No | 13.5 | 14.1 | 17.1 | 17.6 | 17.1 | 16.5 | 16.5 | 16.2 | 16.4 | 16.8 |
| ***Parity*** |  |  |  |  |  |  |  |  |  |  |
| Primiparous | 38.5 | 38.5 | 39.0 | 39.1 | 39.2 | 39.2 | 38.8 | 39.1 | 38.9 | 38.8 |
| Multiparous (2–5) | 59.5 | 59.4 | 59.0 | 58.9 | 58.8 | 58.7 | 59.1 | 58.9 | 59.1 | 59.2 |
| Multiparous (6–12) | 2.0 | 2.1 | 2.0 | 2.0 | 2.0 | 2.0 | 2.1 | 2.0 | 2.1 | 2.0 |
| ***Plurality*** |  |  |  |  |  |  |  |  |  |  |
| Singleton births | 96.9 | 96.9 | 96.9 | 96.9 | 96.8 | 96.9 | 96.8 | 96.9 | 96.8 | 96.8 |
| Multiple births | 3.2 | 3.1 | 3.1 | 3.1 | 3.2 | 3.1 | 3.2 | 3.1 | 3.3 | 3.2 |
| ***Maternal smoking (2007–2013)*** |  |  |  |  |  |  |  |  |  |  |
| Smoked during both first and second trimesters | N/A | N/A | 2.0 | 1.8 | 1.7 | 1.7 | 1.6 | 1.5 | 1.4 | 1.3 |
| ***Prepregnancy body mass index (*kg/m^2^)** |  |  |  |  |  |  |  |  |  |  |
| Underweight (≤ 18.5) | N/A | N/A | 4.4 | 4.2 | 4.1 | 3.9 | 3.9 | 4.0 | 3.9 | 4.0 |
| Normal (18.5–24.9) | N/A | N/A | 51.2 | 50.4 | 49.9 | 49.4 | 49.0 | 48.8 | 48.2 | 48.0 |
| Overweight (25.0–29.9) | N/A | N/A | 25.4 | 25.8 | 25.7 | 25.9 | 26.0 | 25.8 | 26.0 | 25.9 |
| Obese I (30.0–34.9) | N/A | N/A | 11.7 | 12.2 | 12.6 | 12.7 | 12.8 | 12.8 | 13.1 | 13.1 |
| Obese II (35.0–39.9) | N/A | N/A | 4.7 | 4.9 | 5.0 | 5.2 | 5.4 | 5.5 | 5.6 | 5.7 |
| Obese III (≥ 40) | N/A | N/A | 2.6 | 2.7 | 2.8 | 2.9 | 3.0 | 3.2 | 3.3 | 3.3 |

Race and ethnicity results were tabulated using the following racial and ethnic groups: Hispanic, white, Asian, Pacific Islander, African American, multiple races (two or more), American Indian, and other

a. White: individuals identified as white

b. Asian: individuals identified as Asian Indian, Asian (specified or unspecified), Cambodian, Chinese, Filipino, Hmong, Japanese, Korean, Laotian, Thai, and Vietnamese

c. Pacific Islander: individuals identified as Guamanian, Hawaiian, Samoan, and “other Pacific Islander”

d. American Indian: individuals identified as Aleut, American Indian, and Eskimo

e. Other/unknown: individuals identified as “not stated” and “unknown”

N/A = Maternal smoking status and the variables needed to compute prepregnancy body mass index were not recorded during 2005 or 2006

**Table S2. Likelihood of low birth weight listed as adjusted odds ratios (95% confidence intervals) for maternal age for each maternal race and ethnic group, after accounting for confounding effects in California for the period 2005–2014**

| **Maternal age (years)** | **Hispanic** | **White** | **Asian** | **Pacific Islander** | **African American** | **Multiple race** | **American Indian** |
| --- | --- | --- | --- | --- | --- | --- | --- |
| **< 20** | **1.04 (1.01–1.06)** | 1.00 (0.94**–**1.06) | **1.33 (1.20–1.46)** | 0.80 (0.56**–**1.13) | **0.94 (0.88–1.00)** | 1.00 (0.88**–**1.14) | 0.79 (0.58**–**1.09) |
|  | **0.004** | 0.981 | **< .001** | 0.202 | **0.047** | 0.949 | 0.158 |
| **20–24** | Ref | Ref | Ref | Ref | Ref | Ref | Ref |
| **25–29** | **1.11 (1.08–1.13)** | **1.13 (1.09–1.18)** | 0.97 (0.92**–**1.03) | 0.97 (0.78**–**1.21) | **1.13 (1.07–1.18)** | 1.00 (0.91**–**1.11) | 1.16 (0.90**–**1.48) |
|  | **< .001** | **< .001** | 0.314 | 0.792 | **< .001** | 0.991 | 0.256 |
| **30–34** | **1.30 (1.27–1.33)** | **1.31 (1.26–1.36)** | **1.09 (1.03–1.15)** | **1.29 (1.02–1.64)** | **1.33 (1.25–1.40)** | 1.05 (0.94**–**1.17) | **1.65 (1.26–2.17)** |
|  | **< .001** | **< .001** | **0.002** | **0.036** | **< .001** | 0.406 | **< .001** |
| **35–39** | **1.62 (1.58–1.67)** | **1.64 (1.57–1.71)** | **1.30 (1.22–1.37)** | **1.45 (1.09–1.92)** | **1.65 (1.55–1.77)** | **1.37 (1.20–1.56)** | **2.26 (1.63–3.11)** |
|  | **< .001** | **< .001** | **< .001** | **0.010** | **< .001** | **< .001** | **< .001** |
| **40–54** | **2.10 (2.02–2.19)** | **2.17 (2.05–2.29)** | **1.70 (1.59–1.82)** | **2.47 (1.71–3.57)** | **2.14 (1.94–2.36)** | **1.77 (1.48–2.13)** | **3.05 (1.91–4.88)** |
|  | **< .001** | **< .001** | **< .001** | **< .001** | **< .001** | **< .001** | **< .001** |

Results in bold indicate statistical significance (*p* < 0.05)

Ref = Reference group

Multivariable logistic regression models were conducted for each racial and ethnic group, controlling for maternal education, nativity, demographic region, source of prenatal care payment, first-trimester prenatal care initiation, parity, maternal smoking status, and maternal prepregnancy body mass index

**Table S3. Likelihood of low birth weight listed as adjusted odds ratios (95% confidence intervals) for maternal education for each maternal race and ethnic group, after accounting for confounding effects in California for the period 2005–2014**

| **Maternal education** | **Hispanic** | **White** | **Asian** | **Pacific Islander** | **African America** | **Multiple race** | **American Indian** |
| --- | --- | --- | --- | --- | --- | --- | --- |
| **Less than high school diploma** | **1.39 (1.34–1.43)** | **1.64 (1.55–1.73)** | **1.18 (1.10–1.26)** | 1.19 (0.85**–**1.67) | **1.52 (1.41–1.64)** | **1.67 (1.44–1.94)** | **1.59 (1.10–2.30)** |
|  | **< .001** | **< .001** | **< .001** | 0.313 | **< .001** | **< .001** | **0.013** |
| **High school diploma** | **1.34 (1.30–1.38)** | **1.36 (1.32–1.41)** | **1.10 (1.05–1.15)** | 0.95 (0.74**–**1.22) | **1.31 (1.23–1.40)** | **1.32 (1.18–1.48)** | 1.04 (0.75**–**1.46) |
|  | **< .001** | **< .001** | **< .001** | 0.679 | **< .001** | **< .001** | 0.805 |
| **Some college or associate degree** | **1.32 (1.28–1.36)** | **1.28 (1.25–1.32)** | **1.17 (1.13–1.21)** | 0.98 (0.77**–**1.24) | **1.25 (1.18–1.33)** | **1.32 (1.20–1.45)** | 1.08 (0.78**–**1.49) |
|  | **< .001** | **< .001** | **< .001** | 0.838 | **< .001** | **< .001** | 0.640 |
| **Bachelor’s degree or higher** | Ref | Ref | Ref | Ref | Ref | Ref | Ref |

Results in bold indicate statistical significance (*p* < 0.05)

Ref = Reference group

Multivariable logistic regression models were conducted for each racial and ethnic group, controlling for maternal age, nativity, demographic region, source of prenatal care payment, first-trimester prenatal care initiation, parity, maternal smoking status, and maternal prepregnancy body mass index
